# Salivary micro RNAs as biomarkers for oropharyngeal cancer

**DOI:** 10.1002/cam4.6185

**Published:** 2023-06-06

**Authors:** Chameera Ekanayake Weeramange, Kai Dun Tang, Roberto A. Barrero, Gunter Hartel, Zhen Liu, Rahul Ladwa, Julian Langton‐Lockton, Ian Frazer, Lizbeth Kenny, Sarju Vasani, Chamindie Punyadeera

**Affiliations:** ^1^ Saliva and Liquid Biopsy Translational Laboratory Griffith Institute for Drug Discovery (GRIDD) and Menzies Health Institute Queensland (MIHQ) Griffith University Nathan Queensland Australia; ^2^ Menzies Health Institute Queensland (MIHQ) Griffith University Nathan Queensland Australia; ^3^ School of Biomedical Science Centre for Biomedical Technologies Faculty of Health Queensland University of Technology Brisbane Queensland Australia; ^4^ Department of Medical Laboratory Sciences Faculty of Health Sciences The Open University of Sri Lanka Nugegoda Sri Lanka; ^5^ EDA School of Biological Sciences and Biotechnology and Nankai International Advanced Research Institute (Shenzhen Futian) Nankai University Tianjin People's Republic of China; ^6^ eResearch, Academic Division Queensland University of Technology Brisbane Queensland Australia; ^7^ Statistics Unit QIMR Berghofer Medical Research Institute Herston Queensland Australia; ^8^ Department of Otolaryngology Royal Brisbane and Women's Hospital Brisbane Queensland Australia; ^9^ Faculty of Medicine The University of Queensland Herston Queensland Australia; ^10^ Department of Cancer Care Services Princess Alexandra Hospital Woolloongabba Queensland Australia; ^11^ Metro‐North Sexual Health and HIV Service Brisbane Queensland Australia; ^12^ Department of Cancer Care Services Royal Brisbane and Women's Hospital Brisbane Queensland Australia

**Keywords:** biomarkers, human papillomavirus, micro RNA, oropharyngeal cancer, saliva

## Abstract

**Background:**

Despite the rising incidence, particularly of the human papillomavirus (HPV)‐associated fraction of oropharyngeal cancer (OPC), there are no early detection methods for OPC. Considering the close association between saliva and head and neck cancers, this study was designed to investigate salivary micro RNA (miRNAs) associated with OPC, especially focusing on HPV‐positive OPC.

**Methods:**

Saliva was collected from OPC patients at diagnosis and patients were clinically followed up ≤5 years. Salivary small RNA isolated from HPV‐positive OPC patients (*N* = 6), and HPV‐positive (*N* = 4) and negative controls (*N* = 6) were analysed by next‐generation sequencing to identify dysregulated miRNAs. Discovered miRNAs were validated by quantitative PCR using two different assays in a separate cohort of patients (OPC = 91, controls = 92). The relative expression was calculated considering SNORD‐96A as the normalizer. Candidate miRNAs with diagnostic and prognostic potential were evaluated by generalized logistic regression.

**Results:**

A panel consisting of nine miRNAs was identified to have the best diagnostic performance to discriminate HPV‐positive OPC from HPV‐positive controls (AUC‐ validation‐1 = 94.8%, validation‐2 = 98%). Further, a panel consisting of six miRNAs were identified to discriminate OPC from controls regardless of the HPV status (AUC‐ validation‐1 = 77.2%, validation‐2 = 86.7%). In addition, the downregulation of hsa‐miR‐7‐5p was significantly associated with poor overall survival of OPC patients (HR = 0.638). A panel consisting of nine miRNAs were identified for the prediction of the overall survival of the OPC patients (log‐rank test‐*p* = 0.0008).

**Conclusion:**

This study highlights that salivary miRNAs can play an essential role in the detection and prognostication of OPC.

## Graphical abstract

salivary miRNAs can play an essential role in detection and prognostication of OPC

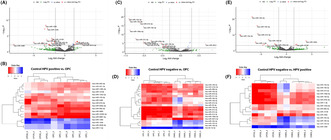

## 1 INTRODUCTION

Micro RNA (miRNA)‐mediated messenger RNA regulation can be identified as one of the most efficient post‐transcriptional gene expression regulation modalities that cells employ. As such, these approximately 22‐nucleotide long, small non‐coding RNAs play an integral role in virtually every cellular process including the development, differentiation and maintenance of cells.^1^ Due to their dynamic engagement in biological pathways, cells maintain a precise composition of miRNAs required depending on the cellular state.^2^, ^3^ However, in disease conditions, this composure often gets disturbed, leading to active or passive changes in miRNA expression. Hence, miRNAs are recognized as robust diagnostic, monitoring and therapeutic targets for a range of disease conditions.^4^, ^5^, ^6^

Cancer is one such disease where dysregulation of miRNA is well‐documented.^7^, ^8^ These changes are not only detectable in the tumour and its microenvironment but can also be detected in surrounding body fluids.^9^, ^10^ As such, liquid biopsy‐based miRNA evaluation can be considered a potential tool for cancer detection. Oropharyngeal cancer (OPC), the disease in focus of this study, is no exception for miRNA expression changes.^11^, ^12^, ^13^ However, liquid biopsy‐based miRNA changes that enable OPC identification are yet to be elucidated. Being in close contact with these tumours, saliva provides an ideal avenue for such biomarker identification.

However, the presence of two clinically and biologically distinct categories of OPC depending on the aetiology, complicates miRNA identification for OPC detection. A subset of OPC is caused by carcinogenic types of human papillomavirus (HPV) and the others are mainly caused by behavioural risk factors such as smoking.^14^ Even though behavioural risk factors accounted for the majority of OPCs in the past, the epidemiological and demographic landscape of OPC has changed considerably over the last two decades due to the alarming rise in HPV‐associated OPC incidence.^15^, ^16^ It was estimated that in year 2020, OPC was responsible for over 98,000 new cases and over 48,000 deaths worldwide.^17^ A significant fraction of these cases, especially in the Western world, is attributed to HPV infection and of these cases 80%–90% are caused by HPV16.^18^, ^19^ In countries such as the USA, OPC has already become the most common HPV‐associated cancer surpassing cervical cancer.^20^

Considering the rising incidence, this study was primarily designed to identify salivary miRNA that can be used as diagnostic and prognostic targets for HPV‐positive OPC. Due to the fact that HPV infection itself causes changes in miRNA expression, this study uses a systematic approach to deduce HPV‐positive‐OPC associated salivary miRNA expression changes accounting for miRNA changes associated with HPV infection.^21^

## 2 MATERIALS AND METHODS

### 2.1 Ethics approval

This study was carried out according to The Code of Ethics of the World Medical Association (Declaration of Helsinki). Informed consent was obtained from all individual participants included in the study. Ethics approval was granted by the Metro South Human Research Ethics Committee [HREC/12/QPAH/381]. The study was also approved by Queensland University of Technology [HREC Nos 1400000617, 1400000641 and 200000043], University of Queensland Medical Ethical Institutional Board [HREC No. 2014000862], and Royal Brisbane and Women's Hospital (RBWH) [HREC/16/QRBW/447].

### 2.2 Participant recruitment

This study includes two groups of OPC patients, HPV‐positive OPC and HPV‐negative OPC and two groups of cancer‐free individuals, HPV‐positive controls and HPV‐negative controls. Sample collection was carried out from 2012 to 2020 in Queensland, Australia. Treatment‐naive OPC patients were recruited from Princes Alexandra Hospital, Royal Brisbane and Women's Hospital, and Logan Hospital. Cancer‐free individuals were recruited from The University of Queensland School of Dentistry, The Queensland University of Technology Health Clinics, Logan Hospital, and Metro‐North Sexual Health and HIV Service. Patients with conditions restricting saliva collection were excluded from the study. OPC patients were clinically followed up for up to 5 years. Survival characteristics were evaluated in terms of overall survival (OS).

### 2.3 Cyclin‐dependent kinase inhibitor 2A (p16) immunohistochemistry

OPC tumour tissues/biopsies were tested for p16 immunohistochemistry by Queensland pathologists as a part of the routine clinical assessment.^19^ CINtec®p16INK4a Histology Kit (E6H4 clone) (Roche MTM Laboratories) was used for the assay and strong diffuse nuclear and cytoplasmic staining present over 70% of tumour tissue was considered as positive for p16.

### 2.4 Saliva collection and processing

Samples were collected according to a previously described method.^22^, ^23^ Briefly, participants were requested to refrain from eating or drinking for at least 1 h prior to saliva collection. Following passive pooling, 2–5 mL of saliva was collected by expectorating into a collection container. Samples were transported on ice and immediately processed before storing at −80°C. Saliva for miRNA isolation was processed by mixing 200 μL of saliva with 800 μL of QIAzol (Qiagen). The remaining saliva was aliquoted and stored as neat saliva.

### 2.5 HPV detection

DNA was isolated from neat saliva using the QIAamp DNA Mini Kit (Qiagen) according to a previously described procedure.^22^ Salivary DNA was tested for HPV16 by quantitative PCR and HPV16‐negative samples were tested for 17 high‐risk types by iPlex MassARRAY (Agena Bioscience). The detailed procedure is described elsewhere.^19^, ^24^

### 2.6 miRNA isolation

miRNA isolation was carried out using the NucleoSpin® miRNA isolation kit (Machnery‐Nagel) according to a previously published protocol with a few minor modifications.^25^, ^26^ The fraction containing 200 μL of saliva with 800 μL of QIAzol (Qiagen) was used. The mixture was vortexed and incubated at room temperature for 5 min. Chloroform (140 μL) was added and the mixture was further incubated for 3 min at room temperature. Following centrifugation at 12,000 × ***g*** for 15 min at 4°C, the clear supernatant was transferred to a separate vial. Ethanol (200 μL) was added to the separated supernatant and large RNAs were isolated by column filtration. Buffer MX (800 μL) was added to the filtrate obtained by the previous step and small RNAs were isolated separately using column filtration. Following three consecutive washing steps with 600 μL of MW1, 700 μL of MW2 and 250 μL of MW1, small RNAs were eluted using 20 μL of ultra‐pure water. The Qubit microRNA assay (Thermo Fisher Scientific) was used for the quantification of isolated miRNA.

### 2.7 Small RNA sequencing

Library preparation and small RNA sequencing were carried out at BGI Genomics (New Territories, Hong Kong). DNBSEQ™ technology involving combinatorial probe‐anchor synthesis (cPAS), linear isothermal rolling‐circle replication and DNA nanoballs (DNB™) technology was used for sequencing. Quantification accuracy was improved by using unique molecular identifiers (UMIs).

### 2.8 miRNA quantification by quantitative reverse transcription PCR (RT‐qPCR)

#### 2.8.1 miScript™ primer PCR assays

Complementary DNA (cDNA) synthesis was carried out using miScript II RT Kit (Qiagen) as per the manufacturer's protocol. Three hundred nanograms of isolated small RNA was used as the input. Briefly, 4 μL of 5x miScript HiSpec Buffer, 2 μL of 10× miScript Nucleics Mix and 2 μL of miScript Reverse Transcriptase Mix were added to the template miRNA and the total volume was adjusted to 20 μL using RNase‐free water. The mixture was incubated for 60 min at 37°C and for a further 5 min at 95°C to heat inactivate the enzymes. The cDNA synthesis process involves polyadenylation of mature miRNAs by poly(A) polymerase and reverse transcription of miRNA using oligo‐dT primers containing a universal tag sequence.

Custom miScript™ primer PCR assays (Qiagen) were used for qPCR amplification of the selected miRNAs. The assay employs a target‐specific forward primer and a universal reverse primer directed towards the universal tag incorporated in the cDNA synthesis step. Five microlitres of 2x QuantiTect SYBR Green PCR master mix, 1 μL of 10x miScript universal primer, 1 μL of 10x miScript primer assay (target‐specific primer) and 6 ng of cDNA adjusted to 3 μL using RNase‐free water were used per each reaction. Amplification was performed using QuantStudio 7 Flex Real‐Time PCR System (Applied Biosystems). PCR conditions were 95°C for 15 min for the initial activation of HotStart Taq DNA polymerase, 40 cycles at 94°C for 15 s, 55°C for 30 s and 70°C for 30 s followed by a melt curve analysis at 95°C for 15 s, 60°C for 1 min and 95°C for 15 s. All samples were tested in duplicate.

#### 2.8.2 miRCURY™ locked nucleic acid (LNA) miRNA PCR assays

The miRCURY® LNA® RT Kit was used for cDNA synthesis and 48 ng of small RNA was used in each reaction. Two microlitres of 5X miRCURY RT reaction buffer, 1 μL of 10X miRCURY RT enzyme mix and 48 ng of small RNA adjusted to 7 μL using RNA‐free water were used per each reverse transcription reaction. The reaction mixture was incubated for 60 min at 42°C and subsequently for 5 min at 95°C for heat inactivation of reverse transcriptase enzyme.

Custom miRCURY™ LNA miRNA PCR assays (Qiagen) were used for qPCR amplification of the selected miRNAs. The assay employs LNA‐based target‐specific forward primer and reverse primer for efficient miRNA quantification. Five microlitres of 2x miRCURY SYBR Green PCR master mix, 0.5 μL of ROX reference dye, 1 μL of PCR primer mix and 1.5 ng of cDNA adjusted to 3.5 μL using RNase‐free water were used for each reaction. PCR amplification was performed using QuantStudio 6 Flex Real‐Time PCR System (Applied Biosystems). PCR conditions were 95°C for 2 min for the heat activation of HotStart Taq DNA polymerase, 40 cycles at 95°C for 10 s, 56°C for 60 s, followed by a melt curve analysis at 95°C for 15 s, 60°C for 1 min and 95°C for 15 s. All samples were tested in duplicate.

### 2.9 Statistical methods

#### 2.9.1 Bioinformatics analysis of sequencing data

The overall quality of raw single‐end reads was assessed using FastQC.^27^ Adaptor sequences and poor‐quality reads were then removed using Trim Galore.^28^ Human miRNA sequences were downloaded from miRbase release 22.1.^29^ High‐quality reads were mapped onto reference human mature and complementary miRNA sequences using bowtie^30^ allowing up to a single mismatch. Feature counts for individual miRNA sequences were determined using SAMtools idxstats.^31^ Differentially expressed miRNAs were determined using DESeq2.^32^

#### 2.9.2 qPCR data analysis

The relative expression (∆Ct) was calculated considering SNORD‐96A as the reference miRNA (126, 295, 302). miRNA expression patterns between groups were investigated using Kruskal–Wallis analysis. Steel‐Dwass multiple comparison method was employed for investigating pair‐wise associations. Performance characteristics of miRNA were evaluated using a generalised logistic regression model using LASSO penalised regression for model selection and Leave‐One‐Out cross‐validation. As two different assays were used for validation 1 and validation 2, different parameter estimates were used to account for assay differences. The cut‐offs for sensitivity and specificity were chosen to balance sensitivity and specificity with a slight preference for better sensitivity in the training set. Survival characteristics were evaluated using Cox proportional hazards modelling with LASSO model selection as before. Statistical analysis was conducted using JMP Pro software version 17.0.0 (SAS Institute).

## 3 RESULTS

### 3.1 Small RNA sequencing

Global profiling of salivary miRNA was carried out by small RNA sequencing in order to discover candidate miRNAs that are capable of discriminating HPV‐positive OPC from cancer‐free individuals. Salivary small RNA isolated from HPV‐positive OPC patients (*N* = 6), HPV‐negative controls (*N* = 6) and HPV‐positive controls (*N* = 4) were considered in the discovery phase.

**FIGURE 1:**
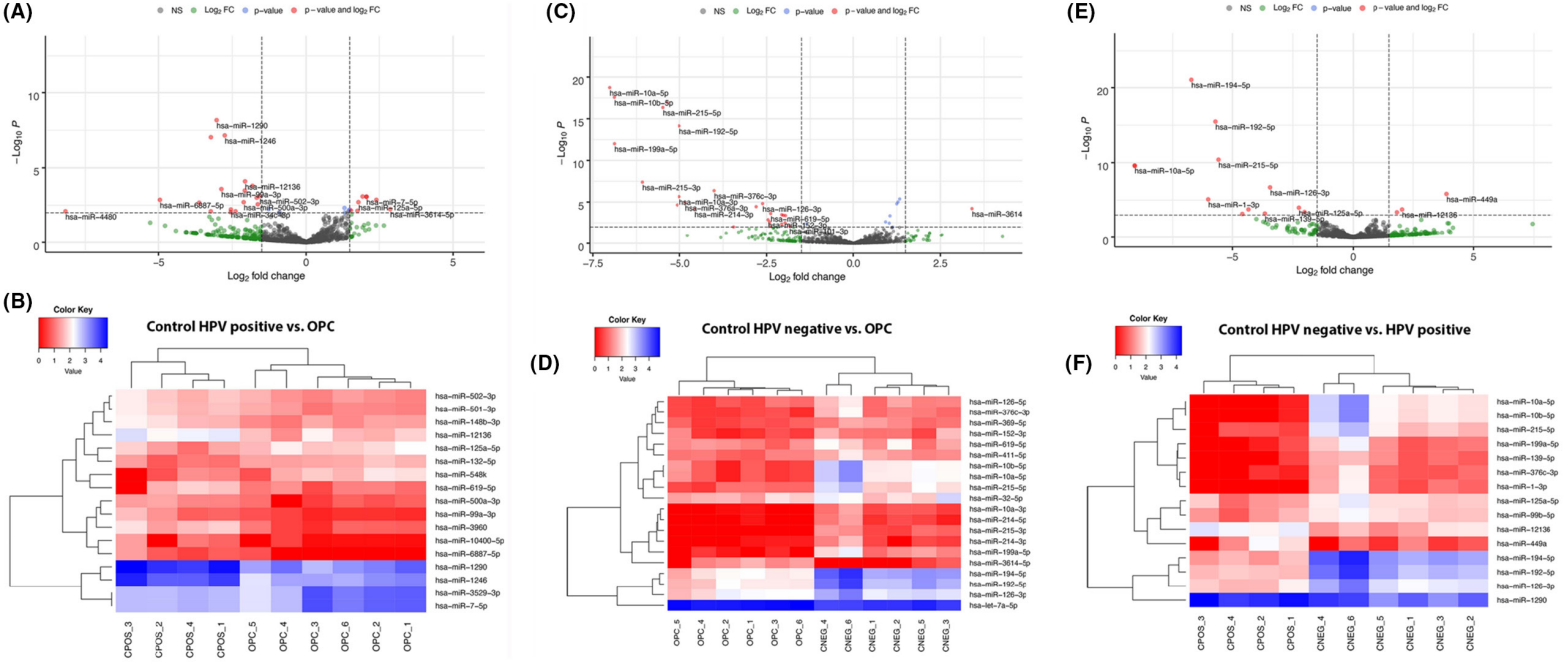
Volcano plot and heat map showing differentially expressed salivary miRNAs (FDR <0.05, logFC >1.5) discovered by small RNA sequencing. (A and B) Control human papillomavirus [HPV]‐positive versus oropharyngeal cancer (OPC) (HPV positive), (C and D) Control HPV‐negative versus OPC (HPV positive), (E and F) Control HPV‐negative versus control HPV‐positive pairwise comparisons.

Differently expressed miRNAs discovered by small RNA sequencing are summarized in Figure 1. DESeq2 algorithm identified 17 differentially expressed miRNAs between HPV‐positive OPC and HPV‐positive controls (Figure 1A,B, Table S1). The strongest association was observed for hsa‐miR‐1290 (*p* = 2.36 × 10^−6^, logFC = −3.030). Similarly, expression differences were identified for 24 miRNAs between HPV‐positive OPC and HPV‐negative controls (Figure 1C,D, Table S2) where hsa‐miR‐10a‐5p (*p* = 1.85 × 10^−19^, logFC = −7.018) was observed to be the leading candidate. A comparison between HPV‐positive controls and HPV‐negative controls identified 15 miRNAs discriminating these groups (Figure 1E,F, Table S3). The strongest association was observed with hsa‐miR‐194‐5p (*p* = 3.20 × 10^−19^, logFC = −6.729).

### 3.2 Validation by quantitative PCR

Following initial screening for the miRNA expression levels in saliva and based on the availability of miScript™ primer PCR assays (Qiagen), 14 miRNAs were selected for further validation by qPCR. The selected miRNAs were tested in a separate cohort of HPV‐positive OPC patients (*N* = 46), HPV‐negative controls (*N* = 46) and HPV‐positive controls (*N* = 16). In order to compare the expression patterns of these miRNAs between HPV‐positive OPC and HPV‐negative OPC, an additional cohort of HPV‐negative OPC samples (*N* = 14) were also tested. Demographic and clinical information of the study participants is listed in Table S4.

Several miRNAs were validated to be markedly dysregulated in the saliva of OPC patients compared to controls (Table 1, Figure S1). miRNA panels that are capable of discriminating OPC patients from controls were identified using a LASSO penalised regression model. Identified miRNA panels were further validated using miRCURY™ LNA miRNA PCR assays (Qiagen) in an additional cohort of HPV‐positive OPC patients (*N* = 31) and HPV‐negative controls (*N* = 30) (Table S5). Similar expression patterns in salivary miRNA were observed in the secondary validation (Table 2, Figure S2).

**TABLE 1:** One‐way Kruskal–Wallis analysis with Steel‐Dwass pairwise comparison of salivary miRNA expression‐miScript™ primer PCR assays.

	Kruskal–Wallis test			Steel‐Dwass multiple comparison procedure *p* values‐valid only if Kruskal–Wallis is significant					
miRNA	Chi‐square	df	Prob>ChiSq	HPV‐positive OPC vs. HPV‐negative OPC	HPV‐positive OPC vs. HPV‐positive control	HPV‐negative OPC vs. HPV‐positive control	HPV‐positive control vs. HPV‐negative control	HPV‐positive OPC vs. HPV‐negative control	HPV‐negative OPC vs. HPV‐negative control
Hsa‐miR‐215‐3p	12.9156	3	0.0250*	0.9137	0.8251	0.8861	0.5764	0.0392*	0.1202
Hsa‐miR‐194‐5p	11.29	3	0.0020*	0.9510	0.0035*	0.0229*	0.0069*	0.7913	0.6835
Hsa‐miR‐126‐3p	6.2508	3	0.0492*	0.9971	0.3511	0.6885	0.9407	0.0583	0.4095
Hsa‐miR‐449a	14.3276	3	0.0024*	0.8048	0.3423	0.1403	1.0000	0.0106*	0.0189*
Hsa‐miR‐199a‐5p	18.228	3	0.0084*	0.9008	0.0193*	0.0576	0.1694	0.3286	0.3992
Hsa‐miR‐3614‐5p	22.1134	3	0.0002*	0.4515	0.0047*	0.0178*	0.5352	0.0257*	0.0170*
Hsa‐miR‐619‐5p	18.2069	3	0.0001*	0.3401	0.0044*	0.0039*	0.1199	0.1541	0.0123*
Hsa‐miR‐07‐5p	10.0129	3	0.0003*	0.0011*	1.0000	0.0018*	1.0000	0.9932	0.0002*
Hsa‐miR‐3529‐3p	16.3767	3	0.0025*	0.5504	0.0052*	0.4522	0.3251	0.0295*	0.9963
Hsa‐miR‐99A‐3p	20.5783	3	0.0101*	0.9737	0.2322	0.3105	0.9858	0.0288*	0.0833
Hsa‐miR‐501‐3p	28.1669	3	<0.0001*	0.0984	0.0062*	0.0034*	0.8251	0.0093*	0.0016*
Hsa‐miR‐1290	23.0891	3	0.0003*	0.9988	0.0890	0.3105	0.9876	0.0005*	0.0445*
Hsa‐miR‐548K	23.5909	3	0.0004*	0.9198	0.0006*	0.0641	0.0856	0.6507	0.0384*
Hsa‐miR‐1246	20.5791	3	0.0002*	0.2221	0.3782	0.2895	0.9998	0.0007*	0.0033*

*p < 0.05/statistically significant.

**TABLE 2:** One‐way Kruskal–Wallis analysis with Steel‐Dwass pairwise comparison of salivary miRNA expression‐Revalidation using miRCURY™ LNA primer PCR assays.

	Kruskal–Wallis Test			Steel‐Dwass multiple comparison procedure *p* values‐valid only if Kruskal–Wallis is significant		
miRNA	Chi‐square	DF	Prob>ChiSq	HPV‐positive OPC vs. HPV‐positive control	HPV‐positive control vs. HPV‐negative control	HPV‐positive OPC vs. HPV‐negative control
Hsa‐miR‐194‐5p	3.8816	2	0.1436	0.1331	0.2751	0.9050
Hsa‐miR‐449a	10.8914	2	0.0043*	0.0187*	0.6040	0.0187*
Hsa‐miR‐3614‐5p	8.6950	2	0.0129*	0.0134*	0.0397*	0.8924
Hsa‐miR‐07‐5p	4.9813	2	0.0829	0.4392	0.1252	0.2942
Hsa‐miR‐3529‐3p	13.0137	2	0.0015*	0.0012*	0.0273*	0.4250
Hsa‐miR‐99A‐3p	40.0824	2	<0.0001*	<0.0001*	0.0131*	<0.0001*
Hsa‐miR‐501‐3p	21.3522	2	<0.0001*	0.0001*	0.5025	0.0009*
Hsa‐miR‐1290	20.4131	2	<0.0001*	0.0004*	0.0185*	0.0062*
Hsa‐miR‐548K	7.6204	2	0.0221*	0.0175*	0.3934	0.2608
Hsa‐miR‐1246	19.8510	2	<0.0001*	<0.0001*	0.0131*	0.0664

*p < 0.05/statistically significant.

### 3.3 Salivary miRNA expression—HPV‐positive OPC versus HPV‐positive controls

Initial qPCR validation identified seven miRNAs with significant salivary expression differences between HPV‐positive OPC and HPV‐positive controls. These miRNAs were downregulated in salivary samples of HPV‐positive OPC patients compared to non‐cancer patients with oral HPV infection. (Table 1, Figure S1). Among them, Hsa‐miR‐548K was the most significantly dysregulated miRNA between these groups (*p* = 0.0006).

**FIGURE 2:**
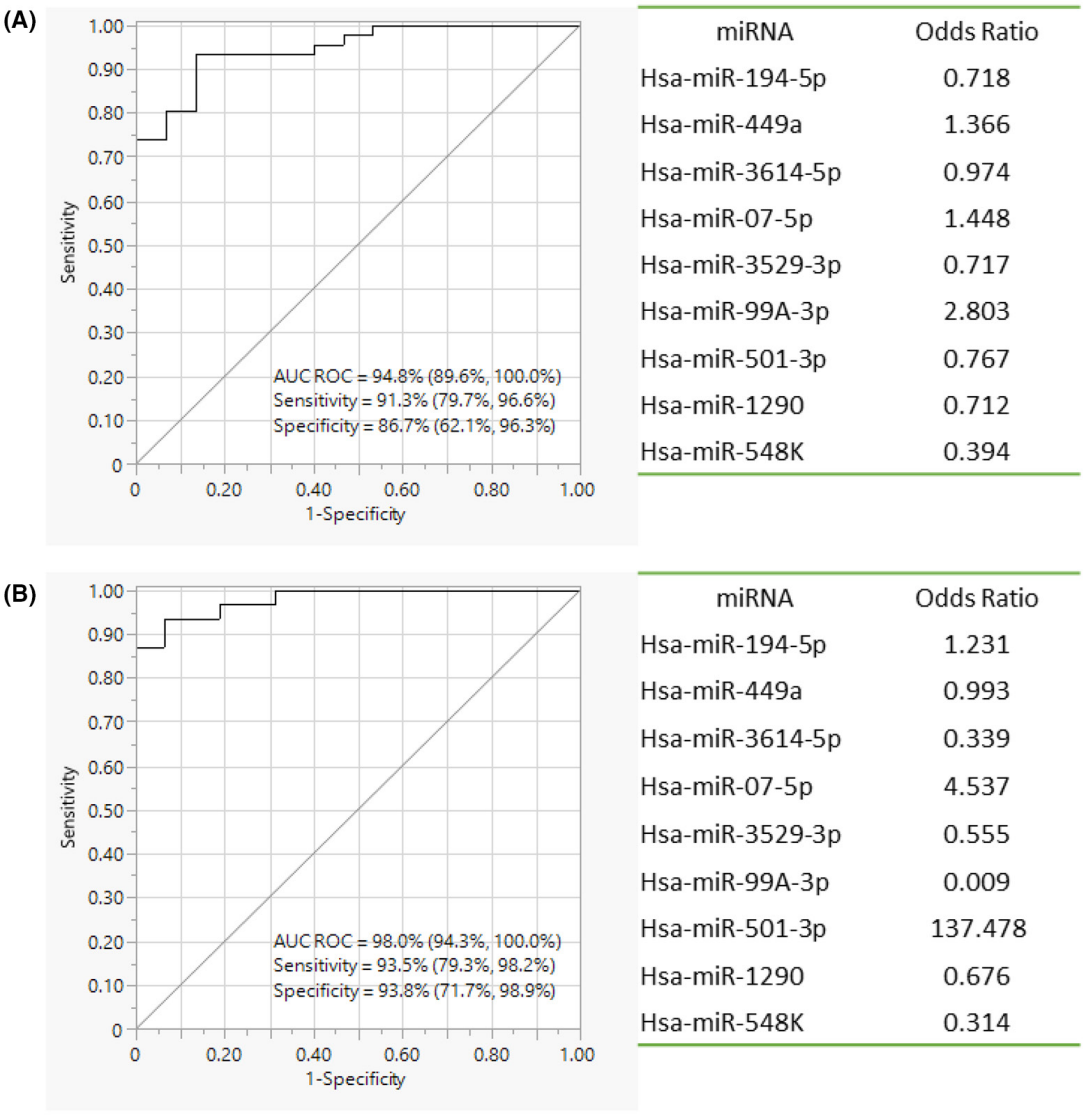
HPV‐positive OPC versus HPV‐positive controls; performance evaluation of miRNA diagnostic panel by ROC analysis and active parameter estimates considered in the panel. (A) qPCR validation 1 by miScript™ primer PCR assays. (B) qPCR validation 2 by miRCURY™ LNA miRNA PCR assays.

Regression modelling identified a salivary diagnostic biomarker panel consisting of nine miRNAs (Hsa‐miR‐194‐5p, Hsa‐miR‐449a, Hsa‐miR‐3614‐5p, Hsa‐miR‐07‐5p, Hsa‐miR‐3529‐3p, Hsa‐miR‐99A‐3p, Hsa‐miR‐501‐3p, Hsa‐miR‐1290, Hsa‐miR‐548K) with the potential to differentiate HPV‐positive OPC patients from HPV‐positive controls (Figure 2A). The panel had a sensitivity of 91.3% (79.7%, 96.6%) and a specificity of 86.7% (62.1%, 96.3%). Receiver operating characteristic (ROC) analysis revealed that the panel has an AUC value of 94.8% (89.6%, 100%).

The efficacy of the panel was retested in a separate cohort of OPC patients using miRCURY™ LNA miRNA PCR assays. However, due to the unavailability of additional HPV‐positive controls, the same (previously tested) cohort was re‐tested using the LNA‐based platform to compare the groups. In the secondary validation, Hsa‐miR‐99A‐3p, which was downregulated in HPV‐positive OPC patients' saliva samples (*p* < 0.0001), demonstrated the most significant differential expression between groups (Table 2, Figure S2). The miRNA panel was able to distinguish HPV‐positive OPC patients from HPV‐positive controls with a sensitivity of 93.5% (79.3%, 98.2%) and a specificity of 93.8% (71.7%, 98.9%). The AUC remained at 98.0% (94.3%, 100%) (Figure 2B).

### 3.4 Salivary miRNA expression–HPV‐negative OPC versus HPV‐negative controls

Although salivary miRNA changes in HPV‐negative OPC patients were not investigated at the discovery phase, several miRNAs considered in the validation phase showed differential expression in the saliva of HPV‐negative OPC patients compared to HPV‐negative controls (Table 2). Eight of these miRNAs were observed to be significantly dysregulated between these groups, including Hsa‐miR‐07‐5p (*p* = 0.0002), displaying the most significant differential expression.

**FIGURE 3:**
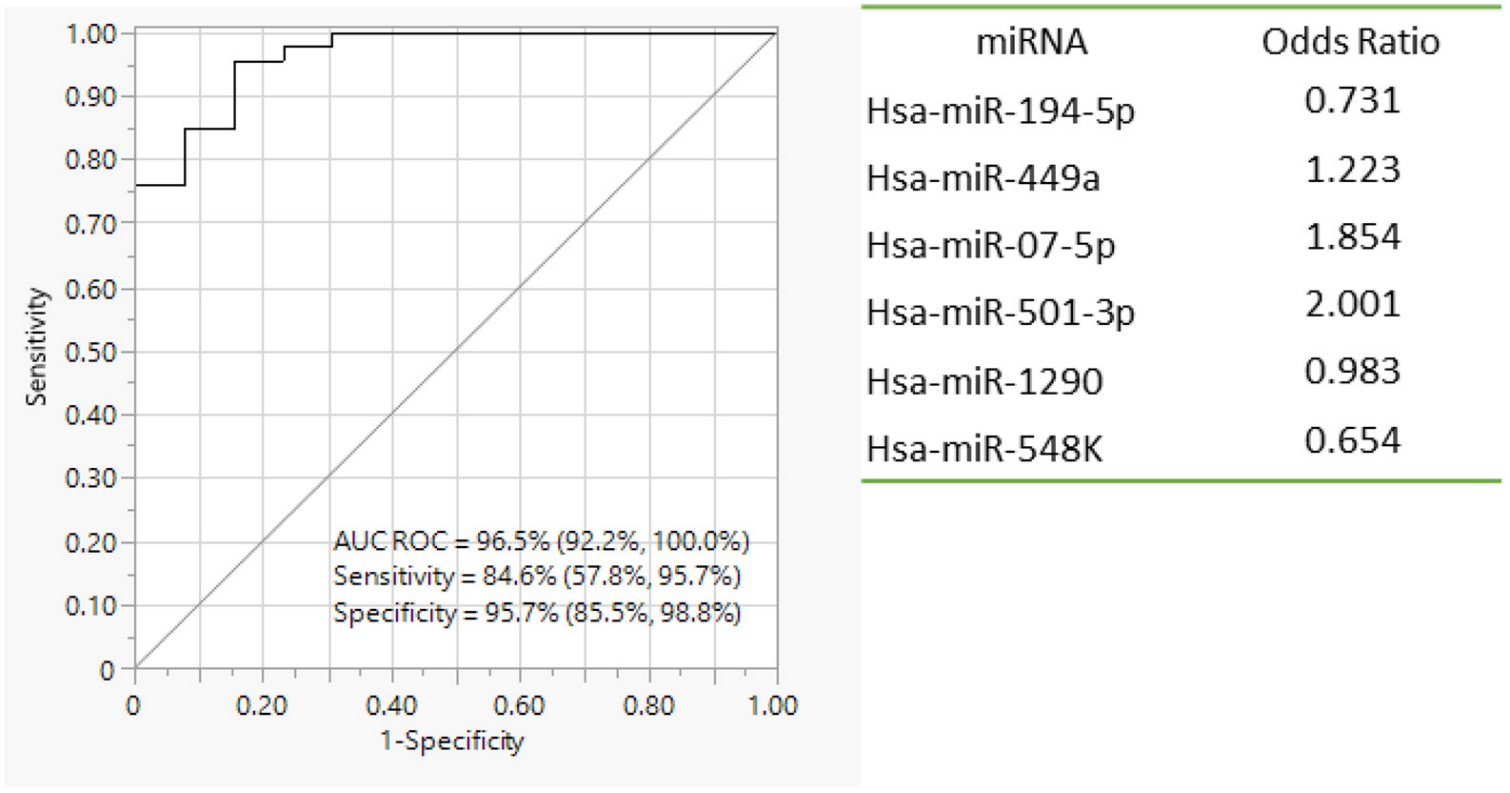
Human papillomavirus (HPV)‐negative oropharyngeal cancer (OPC) versus HPV‐negative controls; performance evaluation of miRNA diagnostic panel by receiver operating characteristic (ROC) analysis and active parameter estimates considered in the panel, qPCR validation 1 by miScript™ primer PCR assays.

**FIGURE 4:**
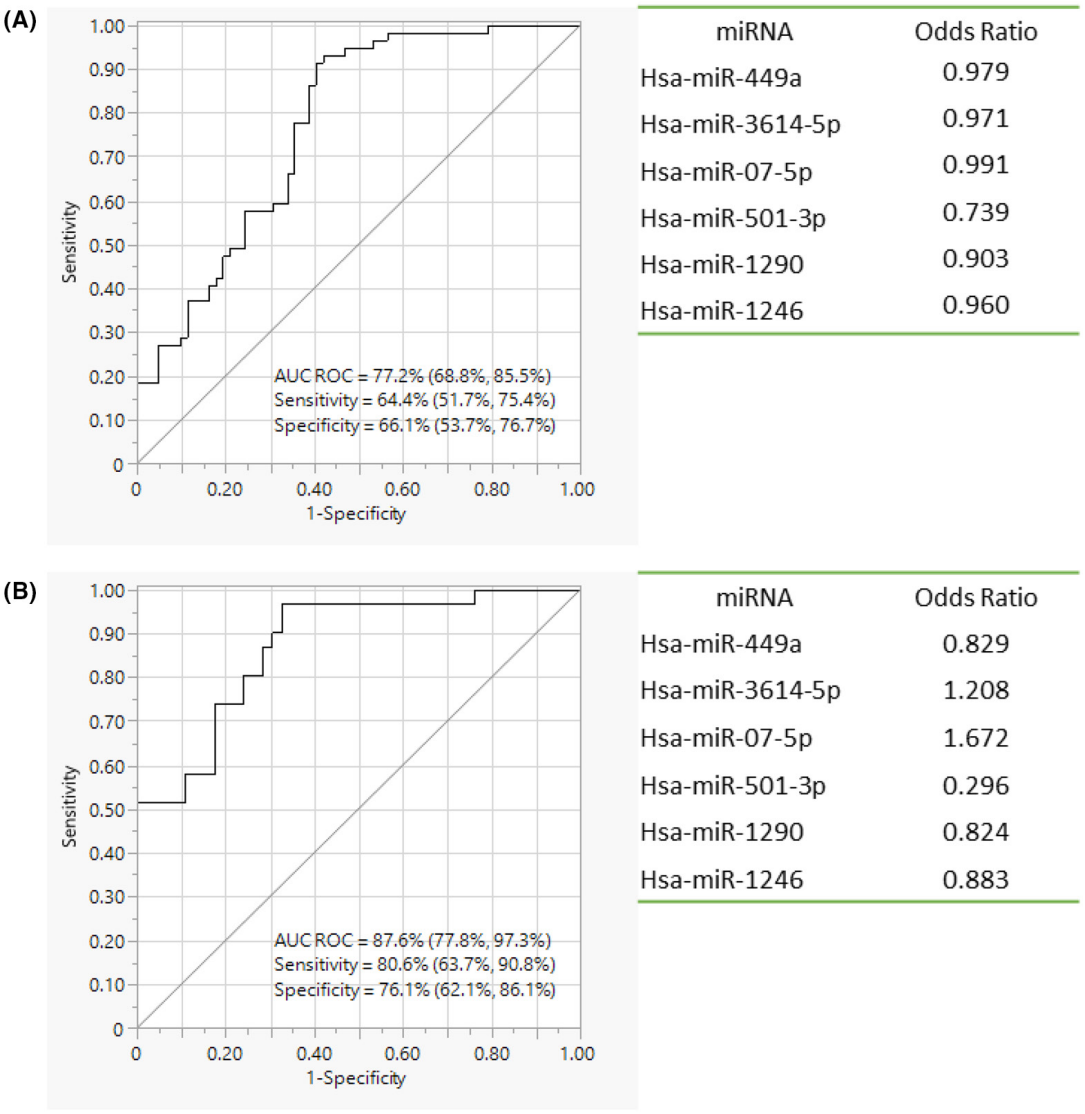
Oropharyngeal cancer (Human papillomavirus [HPV] positive and negative) versus controls (HPV positive and negative); performance evaluation of miRNA diagnostic panel by receiver operating characteristic analysis and active parameter estimates considered in the panel. (A) qPCR validation 1 by miScript™ primer PCR assays. (B) qPCR validation 2 by miRCURY™ LNA miRNA PCR assays.

Regression analysis revealed that a salivary biomarker panel consisting of six miRNAs (Hsa‐miR‐194‐5p, Hsa‐miR‐449a, Hsa‐miR‐07‐5p, Hsa‐miR‐501‐3p, Hsa‐miR‐1290, Hsa‐miR‐548K) could distinguish HPV‐negative OPC patients from controls with a sensitivity of 84.6% (57.8%, 95.7%) and a specificity of 95.7% (85.5%, 98.8%) (Figure 3). The AUC value of the panel was 96.5% (92.2%, 100.0%) (Figure 4A,B). As additional samples of HPV‐negative OPCs were not available, a secondary validation could not be performed. However, leave‐one‐out cross‐validation revealed an AUC of 79.5% (67.7%, 91.3%) for the panel to distinguish HPV‐negative OPC patients from HPV‐negative controls.

### 3.5 Salivary miRNA expression—OPC (HPV positive and negative) versus controls (HPV positive and negative)

Considering that several miRNAs showed similar expression changes in HPV‐positive OPC and HPV‐negative OPC, overall results were combined into two groups (OPC and controls) and evaluated to identify miRNA candidates capable of discriminating OPC from controls irrespective of the HPV status. The regression model identified a panel consisting of six miRNAs (Hsa‐miR‐449a, Hsa‐miR‐3614‐5p, Hsa‐miR‐07‐5p, Hsa‐miR‐501‐3p, Hsa‐miR‐1290, Hsa‐miR‐548K) that is capable of differentiating OPC patients from controls with a sensitivity of 64.4% (51.7%, 75.4%) and a specificity of 66.1% (53.7%, 76.7%). The AUC of the panel was 77.2% (68.8%, 85.5%) (Figure 4A). Secondary validation revealed a sensitivity of 80.6% (63.7%, 90.8%), specificity of 76.1% (62.1%, 86.1%) and an AUC of 87.6% (77.8%, 97.3%) for the assay to distinguish OPC patients from controls (Figure 4B).

### 3.6 Salivary miRNA and OPC patient survival

**FIGURE 5:**
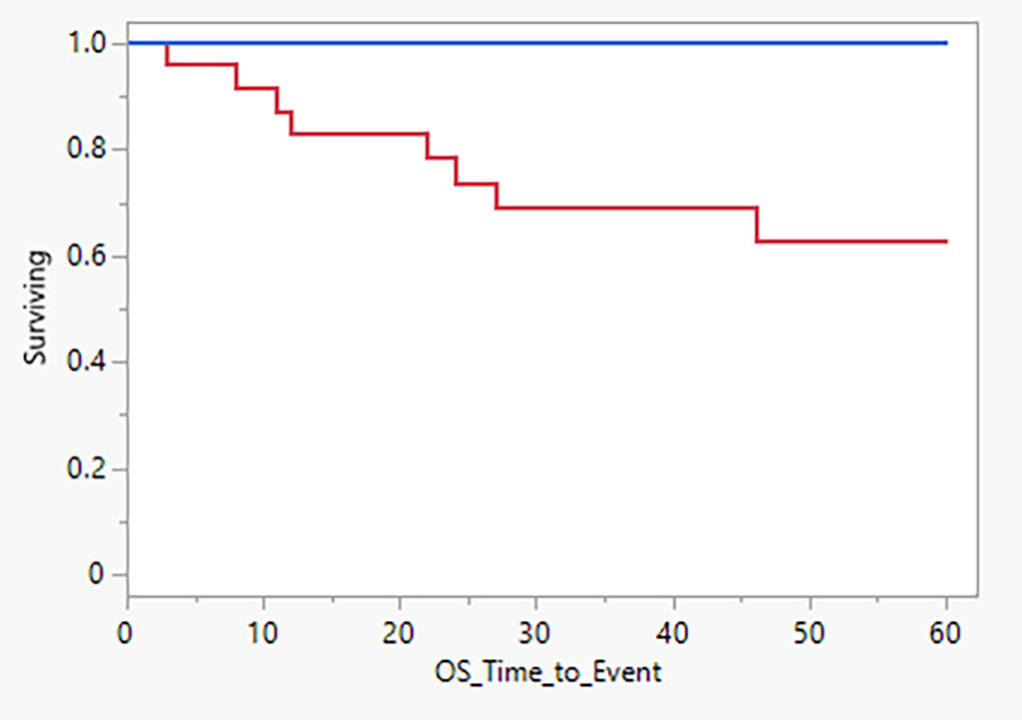
Kaplan–Meier estimate of oropharyngeal cancer (OPC) patient survival by salivary expression of a miRNA panel consisting of Hsa‐miR‐194‐5p, Hsa‐miR‐449a, Hsa‐miR‐199a‐5p, Hsa‐miR‐3614‐5p, Hsa‐miR‐07‐5p, Hsa‐miR‐3529‐3p, Hsa‐miR‐99A‐3p, Hsa‐miR‐501‐3p, Hsa‐miR‐1290, Hsa‐miR‐548K and Hsa‐miR‐1246. Median split cut‐off of linear predictor, cut‐off = 2.787.

The prognostic potential of these miRNAs was investigated in the initial validation cohort. Survival data (up to 5 years) were available for 50 OPC patients and survival characteristics were evaluated in terms of OS. Among the miRNA investigated, hsa‐miR‐07‐5p (HR = 0.638, *p* = 0.001) was identified to have a significant association with OPC prognosis where downregulation was associated with poor prognosis. Cox proportional hazards modelling with LASSO model selection identified that a panel consisting of nine miRNAs (Hsa‐miR‐194‐5p, Hsa‐miR‐449a, Hsa‐miR‐199a‐5p, Hsa‐miR‐3614‐5p, Hsa‐miR‐07‐5p, Hsa‐miR‐3529‐3p, Hsa‐miR‐99A‐3p, Hsa‐miR‐501‐3p, Hsa‐miR‐1290, Hsa‐miR‐548K, Hsa‐miR‐1246) can predict the OS of OPC patients. Kaplan–Meier analysis of the model considering the median split indicated optimal prediction of OPC prognosis (log‐rank test; χ^2^ (1, *N* = 50) = 11.22, *p* = 0.0008) (Figure 5). Revalidation of the panel could not be performed as OS of the secondary validation cohort was 100%.

## 4 DISCUSSION

Considering the rising incidence of HPV‐associated OPC and the lack of early detection methods, the current study was primarily designed to investigate salivary miRNA changes in HPV‐associated OPC and identify candidate miRNAs with diagnostic potential. Traditionally, liquid biopsy‐based cancer biomarker studies underpin biomarker discovery on changes in the tumour tissue and subsequent detection of such changes in body fluids. Conversely, the current study employed a distinct discovery approach where saliva samples of HPV‐associated OPC patients and controls were directly analysed using next‐generation sequencing to discover dysregulated miRNAs in saliva. This approach allows collective identification of detectable salivary miRNA expression changes that are associated with either miRNA changes in the tumour, tumour microenvironment or in the surrounding normal tissue in response to the developing tumour.

The other novel aspect of this study is the investigation of miRNA expression changes that can discriminate HPV‐associated OPC patients from HPV‐positive controls. Viral infections, such as HPV, themselves trigger active and passive changes in miRNA expression.^21^ Even though infection‐associated miRNA changes can play a role in virus‐driven cancer detection, these markers hold a limited value in HPV‐driven cancers as the vast majority of infections spontaneously resolve within 1 to 2 years leaving only a small percentage (approximately 1%–2%) to ever develop HPV driven cancers.^33^, ^34^ By comparing salivary miRNA expression patterns between HPV‐positive OPC patients and HPV‐positive controls, this study reveals that certain salivary miRNA expression changes are capable of effectively discriminating those who have developed HPV‐associated cancers from the infected. In the discovery stage, 17 miRNAs were identified to be differently expressed between these groups. 14 miRNAs were considered for the initial validation and among them, seven were confirmed to be differently expressed between these groups. Furthermore, a panel consisting of nine miRNAs (Hsa‐miR‐194‐5p, Hsa‐miR‐449a, Hsa‐miR‐3614‐5p, Hsa‐miR‐07‐5p, Hsa‐miR‐3529‐3p, Hsa‐miR‐99A‐3p, Hsa‐miR‐501‐3p, Hsa‐miR‐1290, Hsa‐miR‐548K) were identified to have an AUC of 94.8% (89.6%, 100%) for the identification of HPV‐positive OPC patients from HPV‐positive controls. Secondary validation revealed the panel to have an AUC of 98.0% (94.3%, 100%) to distinguish HPV‐positive OPC patients from HPV‐positive controls.

Even though the main objective of the study was to investigate miRNA changes associated with HPV‐positive OPC, several HPV‐negative OPC salivary samples were also evaluated for the 14 miRNA targets that were considered in the validation stage. Among them, eight miRNAs were observed to be differentially expressed between HPV‐negative OPC and HPV‐negative controls. A panel consisting of six miRNAs (Hsa‐miR‐194‐5p, Hsa‐miR‐449a, Hsa‐miR‐07‐5p, Hsa‐miR‐501‐3p, Hsa‐miR‐1290, Hsa‐miR‐548K) could differentiate HPV‐negative OPC from HPV‐negative controls with an AUC of 96.5% (92.2%, 100.0%). However, a secondary validation could not be performed due to the unavailability of additional HPV‐negative OPC samples.

Considering the presence of common OPC‐driven miRNA changes we further evaluated the efficacy of these miRNAs to differentiate OPC from controls regardless of their HPV status. Statistical modelling identified a panel consisting of six miRNAs (Hsa‐miR‐449a, Hsa‐miR‐3614‐5p, Hsa‐miR‐07‐5p, Hsa‐miR‐501‐3p, Hsa‐miR‐1290, Hsa‐miR‐548K) which can distinguish OPC from controls with an AUC of 77.2% (68.8%, 85.5%). Secondary validation revealed an AUC of 87.6% (77.8%, 97.3%) for the panel to distinguish OPC patients from controls.

Although salivary expression patterns of dysregulated miRNAs identified in the current study have not been reported previously, differential expression of these miRNAs has been shown to be associated with different types of cancers. A detailed description of the available literature is summarized in Table S6. The majority of the miRNAs that were observed to be downregulated in the saliva of OPC patients were well‐known tumour suppressors across a wide range of cancers, implying the possibility of underlying functional associations (Table S6). However, there were few exceptions where a downregulation was observed for miRNA that are not well‐known for their tumour suppressor activities. Hsa‐miR‐548K for which salivary expression was downregulated in HPV‐positive and ‐negative OPC compared to their respective controls has not been previously reported to function as a tumour suppressor. Conversely, it has been reported to be frequently overexpressed in oesophageal squamous cell carcinoma (ESCC) and to promote the proliferation and migration of ESCC cells.^35^, ^36^ Similarly, Hsa‐miR‐1290 which was observed to be significantly downregulated in HPV‐negative HNC compared to HPV‐negative controls has also been reported to be over‐expressed in lung and laryngeal cancers tissue and in serum of pancreatic cancer patients.^37^, ^38^, ^39^ However, Hsa‐miR‐1290 has also been reported to be downregulated in plasma of oral squamous carcinoma patients mimicking salivary expression patterns of HPV‐negative OPC patients in the current study.^40^

Furthermore, Hsa‐miR‐1246, which was downregulated in HPV‐negative HNC compared to HPV‐negative controls, is a known tumour‐promoting miRNA in several cancer types and has been reported to be often overexerted in lung cancer and colorectal cancer.^38^, ^41^ In contrast, the study conducted by Bhagirath et al. demonstrates that Hsa‐miR‐1246 expression is downregulated in prostate cancer tissue and upregulation can suppress tumour growth, invasion and migration.^42^ However, they identified that serum Hsa‐miR‐1246 levels to be upregulated, contradicting the tumour expression levels due to exosome‐based selective exportation from cancer cells.^42^ Their findings indicate that miRNA expression pattern in body fluids does not necessarily represent the tumour expression highlighting the fact that our approach to directly characterize liquid biopsy‐based miRNA expression patterns is more applicable from a diagnostic perspective.

The current study also points out that salivary miRNAs have the potential to predict OPC patient outcomes. Cox proportional hazards modelling with LASSO model selection identified that a panel consisting of nine miRNAs (Hsa‐miR‐194‐5p, Hsa‐miR‐449a, Hsa‐miR‐199a‐5p, Hsa‐miR‐3614‐5p, Hsa‐miR‐07‐5p, Hsa‐miR‐3529‐3p, Hsa‐miR‐99A‐3p, Hsa‐miR‐501‐3p, Hsa‐miR‐1290, Hsa‐miR‐548K, Hsa‐miR‐1246) can predict the OS of the OPC patients. However, among the 14 miRNA targets considered only Hsa‐miR‐07‐5p had a significant association with OPC prognosis where lower salivary expression of Hsa‐miR‐07‐5p indicated a poor OS. Hsa‐miR‐07‐5p has been acknowledged as a tumour suppressor by many previous studies for different cancer types.^43^ Moreover, several studies have reported lower expression of Hsa‐miR‐07‐5p to be associated with adverse outcomes in cancers such as colorectal cancer and non‐small cell lung cancer.^44^, ^45^ More importantly, Hsa‐miR‐07 has been shown to promote cisplatin sensitivity in cancers such as lung cancer and gastric cancer.^46^, ^47^ Cisplatin‐based chemotherapy being an important aspect of OPC standard therapy, this could be a possible reason for associations observed in the current study. More so, it is important to highlight that HPV‐positive OPC patients had significantly higher salivary Hsa‐miR‐07‐5p expression levels compared to HPV‐negative OPC and this may also be a contributing factor for enhanced sensitivity for cisplatin therapy in HPV‐positive OPC patients.^14^ However, further investigations are necessary to establish these associations.

## 5 CONCLUSION

While providing insight into salivary miRNA expression changes associated with HPV‐positive and ‐negative OPC, this study proposes that these changes can be used to detect OPC and predict patient outcomes in advance. More importantly, this study reveals that there are detectable salivary miRNA changes in HPV‐positive OPC patients compared to HPV‐infected individuals highlighting a novel approach for early detection of HPV‐driven OPC by coupling salivary HPV detection and miRNA evaluation. Due to its non‐invasive nature and being one of the most convenient specimens to collect, saliva is an ideal matrix for screening purposes. As such, we believe that the proposed biomarker panels can play a pivotal role in the detection and management of OPC.

## 6 LIMITATIONS OF THE STUDY

Several miRNA expression patterns identified by next‐generation sequencing (discovery phase) could not be replicated in the validation phase possibly due to the limited number of samples considered in the discovery phase. Expression patterns in the discovery and validation phase for hsa‐miR‐3529‐3p and hsa‐miR‐548k between HPV‐positive OPC and HPV‐positive controls and hsa‐miR‐3614‐5p between HPV‐positive OPC and HPV‐negative controls did not align. In such instances, expression patterns observed in the validation phase were considered due to the larger sample size. Secondary validation could not be performed for the miRNA panel discriminating HPV‐negative OPC and HPV‐negative controls due to sample unavailability. Further, due to the unavailability of HPV‐positive control samples same cohort was retested using miRCURY™ LNA miRNA PCR assays and reconsidered for the secondary validation.

## AUTHOR CONTRIBUTIONS

**Chameera Ekanayake Weeramange:** Data curation (lead); formal analysis (lead); investigation (lead); methodology (equal); visualization (lead); writing – original draft (lead). **Kai Dun Tang:** Data curation (supporting); formal analysis (supporting); investigation (supporting); methodology (supporting); supervision (supporting); validation (supporting); visualization (supporting); writing – review and editing (supporting). **Roberto A. Barrero:** Data curation (equal); formal analysis (equal); methodology (equal); software (equal); writing – review and editing (equal). **Gunter Hartel:** Data curation (equal); formal analysis (equal); methodology (equal); software (equal); writing – review and editing (equal). **Zhen Liu:** Data curation (equal); formal analysis (equal); resources (equal); writing – review and editing (equal). **Rahul Ladwa:** Formal analysis (equal); methodology (equal); resources (equal); visualization (equal); writing – review and editing (equal). **Julian Langton‐Lockton:** Formal analysis (equal); investigation (equal); resources (equal); supervision (equal); writing – review and editing (equal). **Ian Frazer:** Formal analysis (equal); investigation (equal); supervision (equal); writing – review and editing (equal). **Lizbeth Kenny:** Data curation (equal); formal analysis (equal); investigation (equal); methodology (equal); resources (equal); supervision (equal); writing – review and editing (equal). **Sarju Vasani:** Data curation (equal); formal analysis (equal); investigation (equal); methodology (equal); resources (equal); writing – review and editing (equal). **Chamindie Punyadeera:** Conceptualization (lead); funding acquisition (lead); methodology (lead); project administration (lead); resources (lead); supervision (lead); validation (lead); visualization (equal); writing – review and editing (lead).

## FUNDING INFORMATION

This work was supported by Royal Brisbane Women's Hospital Foundation, Cancer Australia Grant (APP1145657) and National Health and Medical Research Council Grant (APP 2012560) funding available for Chamindie Punyadeera. Chameera Ekanayake Weeramange was supported by a scholarship from the University Grants Commission, Sri Lanka, and Queensland University of Technology, Australia.

## CONFLICT OF INTEREST STATEMENT

The authors confirm that there is no conflict of interest to declare.

## Supporting information


Figure S1.

Figure S2.

Table S1.

Table S2.

Table S3.

Table S4:

Table S5:

Table S6:

## Data Availability

The datasets used and/or analysed during the current study are available from the corresponding author on reasonable request.
