# Supplementary figures and images for: Salivary micro RNAs as biomarkers for oropharyngeal cancer
Source: Cancer Med. 2023 Jun 6;12(14):15128–40. doi: 10.1002/cam4.6185 (PMC10417169; doi:10.1002/cam4.6185)

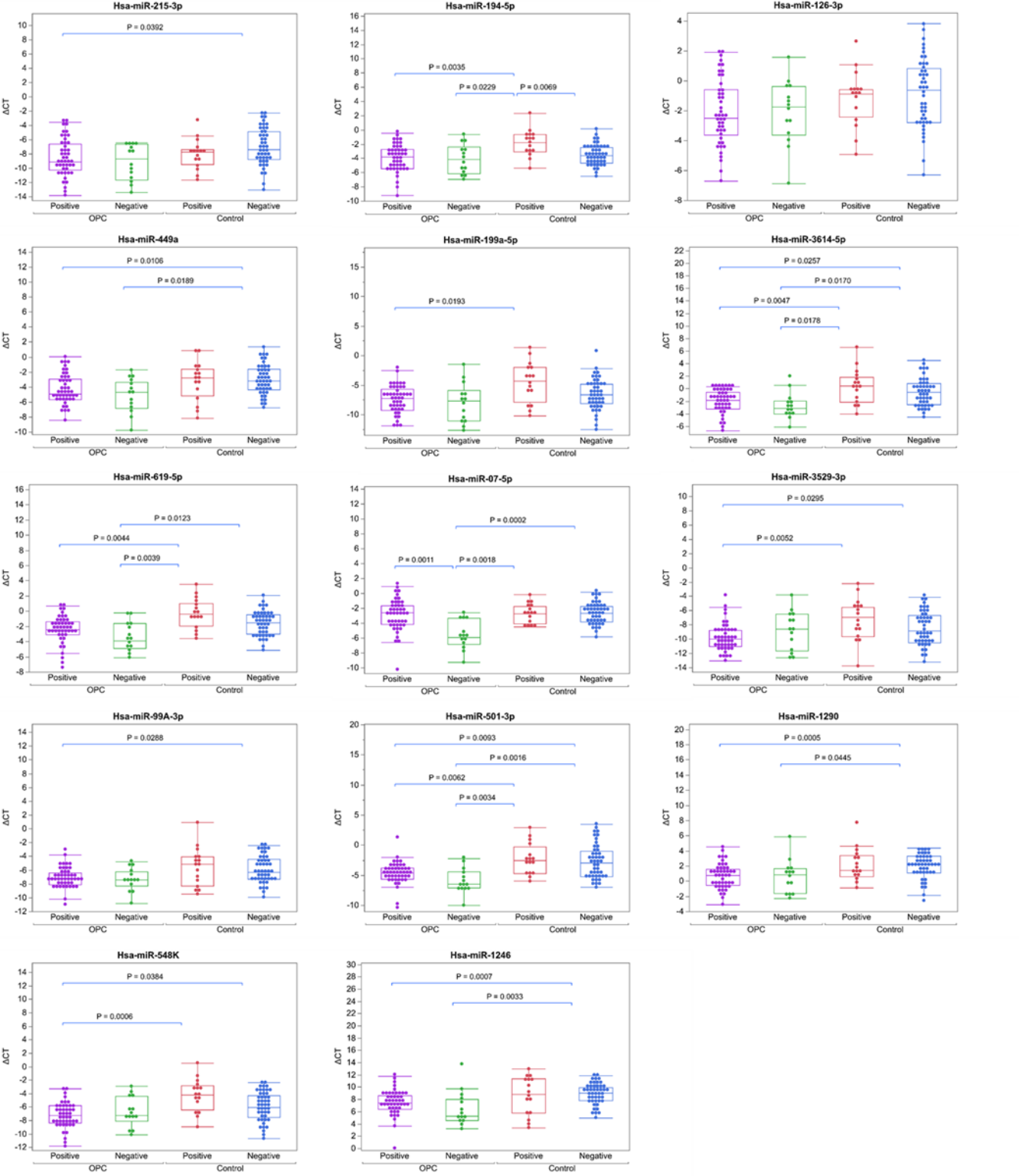

Supplement: Supplementary file 1 — Figure S1. [file CAM4-12-15128-s004.pdf]

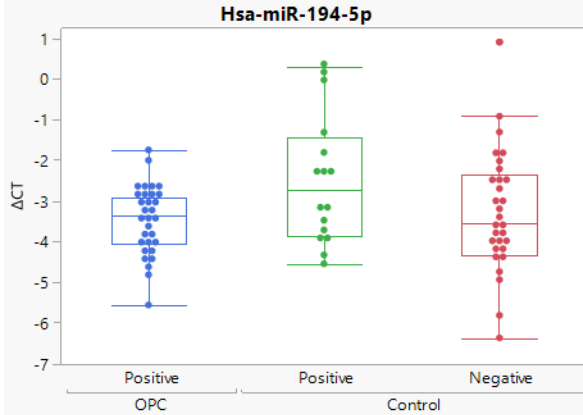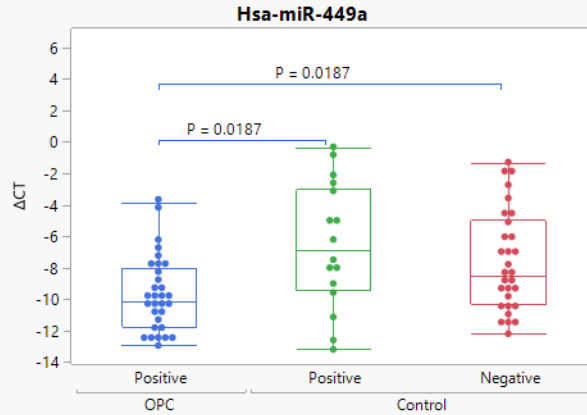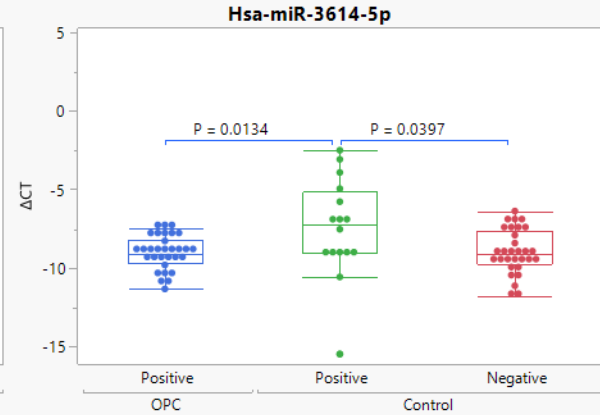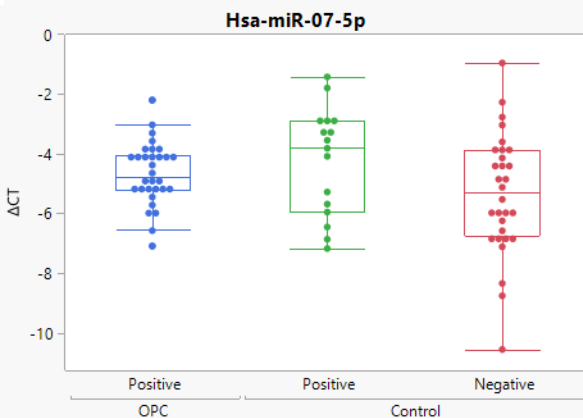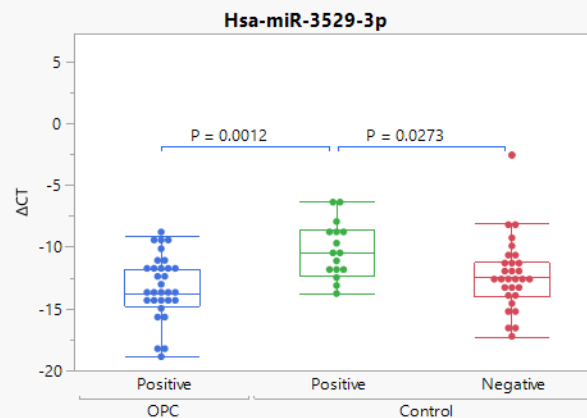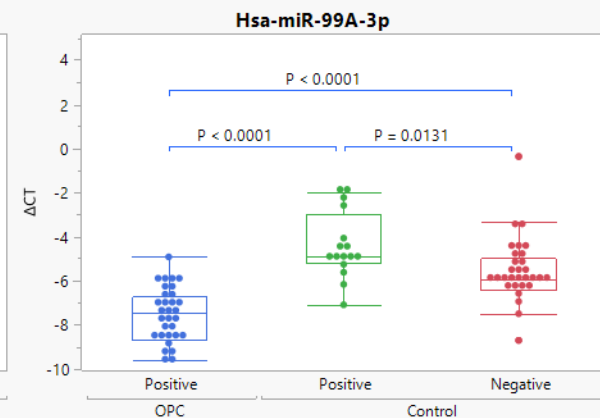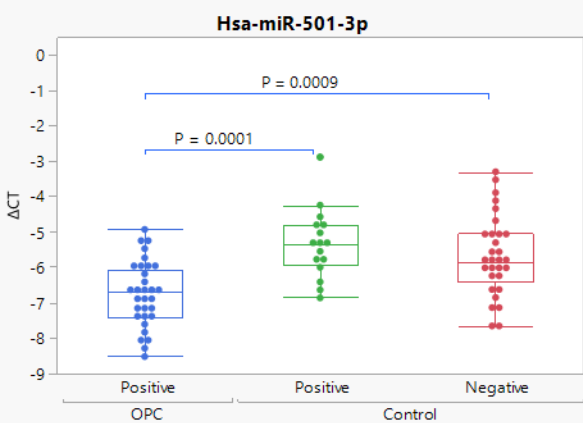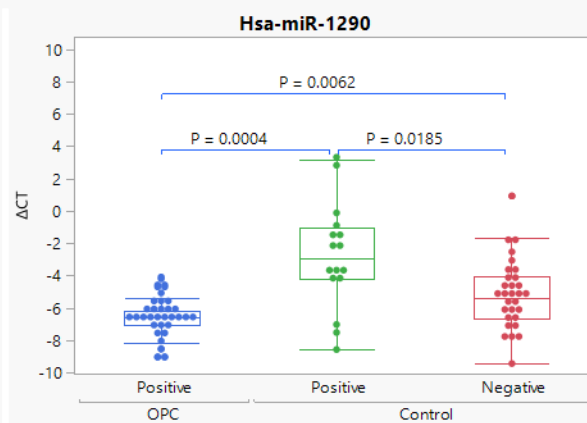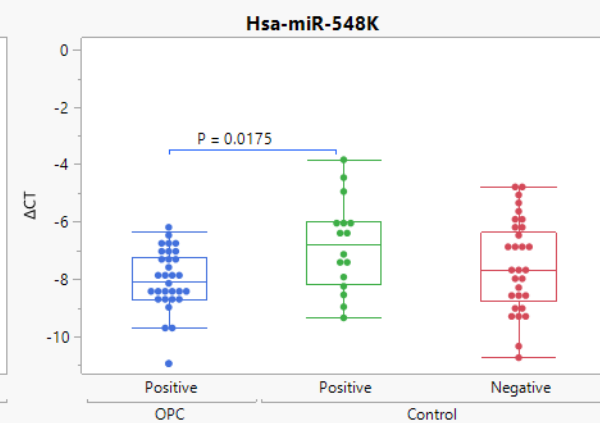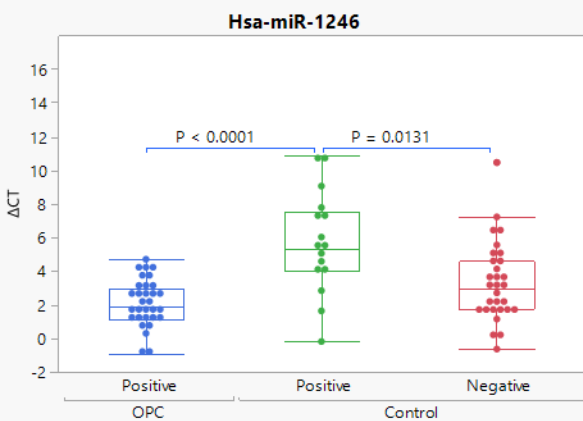

Supplement: Supplementary file 2 — Figure S2. [file CAM4-12-15128-s006.pdf]
