# Supplementary material for: Salivary micro RNAs as biomarkers for oropharyngeal cancer
Source: Cancer Med. 2023 Jun 6;12(14):15128–40. doi: 10.1002/cam4.6185 (PMC10417169; doi:10.1002/cam4.6185)
Supplement: Supplementary file 6 — Table S4: [file CAM4-12-15128-s001.docx]

Table S4: Demographic and clinical characteristics of participants considered for qPCR validation using miScript™ primer PCR Assays

|  |  | HPV-positive OPC (N=46) | HPV-negative OPC (N=14) | HPV-positive controls (N=16) | HPV-negative controls (N=46) |
| --- | --- | --- | --- | --- | --- |
| Age | <= 55 | 8 (17.4%) | 3 (21.4%) | 8 (50.0%) | 17 (37.0%) |
|  | 56 - 65 | 21 (45.7%) | 7 (50.0%) | 4 (25.0%) | 14 (30.4%) |
|  | >= 66 | 17 (37.0%) | 4 (28.6%) | 4 (25.0%) | 15 (32.6%) |
|  | Mean (SD) | 61.87 (8.24) | 59.64 (7.92) | 56.56 (12.73) | 58.48 (12.72) |
| Gender | Male | 44 (95.7%) | 10 (71.4%) | 13 (81.2%) | 39 (84.8%) |
|  | Female | 2 (4.3%) | 4 (28.6%) | 3 (18.8%) | 7 (15.2%) |
| OPC site | Tonsil | 27 (58.7%) | 4 (28.6%) | - | - |
|  | BOT | 13 (28.3%) | 4 (28.6%) |  |  |
|  | Tonsil & BOT | 5 (10.9%) | 0 |  |  |
|  | Other | 1 (2.2%) | 6 (42.9%) |  |  |
| AJCC stage (8^th^ Edition) | Stage 01 | 4 (8.7%) | 0 | - | - |
|  | Stage 02 | 28 (60.9%) | 1 (7.1%) |  |  |
|  | Stage 03 | 12 (26.1%) | 5 (35.7%) |  |  |
|  | Stage 04 | 0 | 6 (42.9%) |  |  |
|  | Not Available | 2 (4.3%) | 2 (14.3%) |  |  |
