# Supplementary material for: Salivary micro RNAs as biomarkers for oropharyngeal cancer
Source: Cancer Med. 2023 Jun 6;12(14):15128–40. doi: 10.1002/cam4.6185 (PMC10417169; doi:10.1002/cam4.6185)
Supplement: Supplementary file 7 — Table S5: [file CAM4-12-15128-s008.docx]

Table S5: Demographic and clinical characteristics of participants considered for qPCR validation using miRCURY ™ LNA primer PCR assays. Due to the unavailability of additional HPV-positive controls, the same cohort used for validation 1 was re-tested using the LNA-based platform to compare the groups.

|  |  | HPV positive OPC (N=31) | HPV positive controls (N=16) | HPV negative controls (N=30) |
| --- | --- | --- | --- | --- |
| Age | <= 55 | 9 (30.0%) | 8 (50.0%) | 6 (20.0%) |
|  | 56 - 65 | 13 (43.3%) | 4 (25.0%) | 7 (23.3%) |
|  | >= 66 | 8 (26.7%) | 4 (25.0%) | 17 (56.7%) |
|  | Mean (SD) | 61.23 (9.77) | 56.56 (12.73) | 66.33 (10.33) |
| Gender | Male | 25 (80.6%) | 13 (81.2%) | 28 (93.3%) |
|  | Female | 2 (6.5%) | 3 (18.8%) | 2 (6.7%) |
| OPC site | Tonsil | 21 (67.7%) | - | - |
|  | BOT | 10 (32.3%) |  |  |
| AJCC stage (8^th^ Edition) | Stage 01 | 21 (67.7%) | - | - |
|  | Stage 02 | 9 (29.0%) |  |  |
|  | Stage 03 | 1 (3.2%) |  |  |
