# Supplementary material for: Salivary micro RNAs as biomarkers for oropharyngeal cancer
Source: Cancer Med. 2023 Jun 6;12(14):15128–40. doi: 10.1002/cam4.6185 (PMC10417169; doi:10.1002/cam4.6185)
Supplement: Supplementary file 8 — Table S6: [file CAM4-12-15128-s002.docx]

Table S6: Associations reported by previous studies for identified miRNA candidates

| **miRNA** | **Finding of the current study** | **Findings of previous studies** | |
| --- | --- | --- | --- |
|  |  | **Study** | **Findings** |
| Hsa-miR-215-3p | Down-regulated in HPV positive OPC compared to HPV negative controls | Hou et al., 2015 [1] | (Hsa-miR-215) Downregulated in non-small cell lung cancer (NSCLC) tissue and cell lines. Suppress cell proliferation, migration, and invasion. Targets Zinc finger E-box-binding homeobox 2 (ZEB2). |
|  |  | Ge et al., 2016 [2] | (Hsa-miR-215) Downregulated in epithelial ovarian cancer tissue and cell lines. Suppress cell proliferation and promote apoptosis and chemotherapy drug response. Regulates X-linked inhibitor of apoptosis. |
|  |  | LEBLANC et al., 2018 [3] | (Hsa-miR-215) Downregulated in breast cancer tissue. Suppress cell proliferation and migration. |
|  |  | Tang et al., 2019 [4] | Targets Forkhead box protein M1 (FOXM1) suppressing growth and invasion of colorectal cancer (CRC) cells. |
|  |  | Deng et al., 2014 [5] | (Hsa-miR-215) Upregulated in gastric cancer tissue. Targets retinoblastoma tumor-suppressor gene 1 (RB1) promoting cell proliferation. |
| Hsa-miR-194-5p | Down-regulated in HPV positive OPC and HPV negative OPC compared to HPV positive controls  Up-regulated in HPV positive controls compared to HPV negative controls | Cai et al., 2021 [6] | Downregulated in esophageal cancer. Suppress proliferation invasion and self-renewal by targeting protein regulator of cytokinesis 1 (PRC1). |
|  |  | Wang et al., 2019 [7] | Downregulated in Hepatocellular carcinoma (HCC) tissue. Function as a tumor suppressor by targeting forkhead box A1 (FOXA1). |
|  |  | Wang et al., 2021 [8] | Downregulated in pancreatic cancer tissue. Function as a tumor suppressor by inhibiting proliferation, invasion, migration, and PD-L1 expression. |
|  |  | Bai et al., 2020 [9] | Downregulated in ovarian cancer tissues. Target Insulin-like growth factor 1 receptor (IGF1R) and Protein-Tyrosine Phosphatase Receptor-Type F Polypeptide-Interacting Protein (PPFIBP). Inhibit proliferation, invasion, and migration. |
|  |  | Wang et al., 2020 [10] | Downregulated in Cisplatin resistant oral squamous cell carcinoma (OSCC). Suppress PVT1 associated cisplatin resistance and cell proliferation. |
| Hsa-miR-449a | Down-regulated in HPV Positive OPC and HPV negative OPC compared to HPV Negative Controls | Veena et al., 2020 [11] | Downregulated in cervical cancer cell lines and tumor tissue. Targets PACS-1 and suppress S phase of the cell cycle and cell growth with improved DNA damage response. |
|  |  | Ye et al., 2014 [12] | Downregulated in type II endometrial cancer tissues. Suppress cell proliferation, invasion, and clonogenic survival. |
|  |  | Bach et al., 2018 [13] | Downregulated in Epidermal growth factor receptor tyrosine kinase inhibitor-resistant NSCLC cells. Induce phosphatase and tensin homolog (PTEN) and suppress Nicotinamide N-methyltransferase (NNMT) and cell growth. |
|  |  | Noonan et al., 2009 [14] | Downregulated in prostate cancer tissue. Target Histone deacetylases1 (HDAC-1) and suppress growth. |
|  |  | Lu et al., 2019 [15] | Up-regulated levels in HNSCC tissue indicate a poor prognosis. |
| Hsa-miR-199a-5p | Down-regulated in HPV positive OPC compared to HPV positive controls | Lajer et al., 2012 [16] | Downregulated in HPV positive HNC tissue and cervical cancer tissue and reported to be a core HPV miRNA. |
|  |  | Tsukigi et al., 2012 [17] | Expression is decreased in certain renal cancer tissue samples. Targets Glycogen synthase kinase-3β (GSK-3β) and suppress growth. |
|  |  | Ye et al., 2015 [18] | Down-regulated in CRC. Inhibit invasion, migration, and cell proliferation. |
|  |  | Byrnes et al., 2016 [19] | Downregulated in esophageal cancer cell lines. Target mitogen-activated protein kinase kinase-11 (MAP3K11) and suppress cell proliferation. |
|  |  | Sakaguchi et al., 2017 [20] | (miR-199 family) Downregulated in bladder cancer. Inhibit cell migration and invasion. |
| Hsa-miR-3614-5p | Down-regulated in HPV positive OPC and HPV negative OPC compared to HPV positive controls and HPV negative controls | Han et al., 2021 [21] | Downregulated in CRC tissue. Expression is inversely associated with overall survival. Improve response of certain immune cells to CRC. |
|  |  | Li et al., 2020 [22] | Downregulated in NSCLC tissues. Target phosphoglycerate mutase 1 (PGAM1) in a hypoxia-dependent manner and suppress cell proliferation and invasion. |
|  |  | Yin and Tong, 2021 [23] | Interacts with ankyrin repeat domain 1 (ANKRD1) and suppress invasion and proliferation of osteosarcoma cells |
| Hsa-miR-619-5p | Down-regulated in HPV positive OPC and HPV negative OPC compared to HPV positive controls  Down-regulated in HPV negative OPC compared to HPV negative controls | Song et al., 2021 [24] | Downregulated in OSCC cell lines and tumor tissue. Suppress invasion, migration, and cell proliferation of cisplatin-resistant OSCC cells by targeting ATXN3. |
|  |  | Zheng et al., 2020 [25] | Suppress cell proliferation and invasion in HNSCC cell lines. |
|  |  | Zhang et al., 2019 [26] | Target DNA polymerase eta (PolH) in NSCLC and bladder cancer suppressing PolH mediated cell growth and cisplatin resistance. |
|  |  | Shkurnikov et al., 2017 [27] | Serum level increases as the stage advances in prostate cancer |
|  |  | Kim et al., 2020 [28] | Upregulated in NSCLC tissues and plasma. Target RCAN1.4 and promote metastasis and tumor angiogenesis. |
| Hsa-miR-07-5p | Down-regulated in HPV negative OPC compared to HPV negative controls and HPV positive controls | Li et al., 2019 [29] | Downregulated in NSCLC tissues and cell lines. Down-regulation is associated with poor prognosis. Suppress cell proliferation and promote apoptosis and G0/G1 arrest. |
|  |  | Yin et al., 2018 [30] | Downregulated in glioblastoma tissues and cell lines. Target special AT rich sequence binding protein 1 (SATB1) and inhibit invasion and migration and promote apoptosis. |
|  |  | Dong et al., 2019 [31] | Downregulated in colorectal cancer tissue and cell lines. Suppress cell proliferation and migration. Target Krüppel‑like factor 4 (KLF4). Decreased expression indicates poor overall survival. |
|  |  | Jung et al., 2012 [32] | (Hsa-miR-07) Upregulated in keratinizing oral cancer tissue. Target reversion inducing cysteine rich protein with kazal motifs (RECK). |
| Hsa-miR-3529-3p | Down-regulated in HPV positive OPC compared to HPV positive and negative controls | Weng et al., 2021 [33] | Downregulated in liver cancer cell lines and patients’ serum. Suppress invasion and migration. Regulate forkhead box protein C2 (FOXC2). |
|  |  | Zhang et al., 2018 [34] | Down-regulated in HPV positive HNSCC tissue compared to HPV negative HNSCC tissue |
|  |  | Kinget et al., 2021 [35] | Regulate HIF-2α and VEGFR1. Elevated expression is associated with poor prognosis for patients treated with VEGFR tyrosine kinase inhibitors. |
|  |  | Shang et al., 2021 [36] | Upregulated in radiotherapy resistant CRC cells. |
| Hsa-miR-99A-3p | Down-regulated in HPV positive OPC compared to HPV negative controls | Lajer et al., 2012 [16] | Downregulated in HPV positive tonsillar squamous cell carcinoma tissue (TSCC), HPV negative TSCC and cervical cancer tissue. |
|  |  | Okada et al., 2019 [37] | Downregulated in HNSCC tissue and cell lines. Lower expression indicates poor prognosis. Inhibit invasion and migration. |
|  |  | Qi et al., 2021 [38] | Downregulated in HNSCC tissue. Associated with TNM stage where HNSCC patients with lower expression have advanced-stage cancers |
|  |  | Shinden et al., 2021 [39] | Downregulated in breast cancer tissues. Inhibit cell growth in breast cancer cells and cell lines. Regulate expression of several genes including FAM64A. |
| Hsa-miR-501-3p | Down-regulated in HPV positive OPC and HPV negative OPC compared to HPV positive controls and HPV negative controls | Luo et al., 2018 [40] | Downregulated in metastatic HCC cell lines and patients with recurrence and metastasis. Suppress proliferation, invasion, migration, and epithelial-mesenchymal transition. Target Lin-7 homolog A (LIN7A). |
|  |  | He et al., 2021 [41] | Downregulated in renal cell carcinoma tissues and cell lines. Suppress proliferation by G1 phase arrest. Regulate WTAP. |
|  |  | Lu et al., 2020 [42] | Downregulated in NSCLC tissue and cell lines. Inhibit cell proliferation and invasion by repressing RAP1A. |
|  |  | Wu et al., 2019 [43] | Upregulated in CRC tissues. Promote cell proliferation and sphere formation. Regulate Wnt/β-catenin signaling pathway. |
|  |  | Sanches et al., 2018 [44] | Upregulated in cervical cancer tissue. promotes cell proliferation, invasion, and migration by downregulating Cylindromatosis (CYLD) |
| Hsa-miR-1290 | down-regulated in HPV negative HNC compared to HPV negative controls | Nakashima et al., 2019 [45] | Downregulated in plasma of OSCC patients. Lower plasma miR-1290 level indicates poor prognosis. |
|  |  | Zhang et al., 2016 [46] | Upregulated in lung cancer CD166+ tumor initiating cells, tumor tissue, and patients’ serum. Promote tumorigenesis and metastatic potential. Inhibition suppress sphere-formation and tumor growth. |
|  |  | Yao et al., 2013 [47] | Up-regulate following demethylation treatment in HPV positive cervical cancer cell lines. |
|  |  | Zhang et al., 2013 [48] | Upregulated in radiotherapy resistant cervical cancer cell lines compared to radiotherapy sensitive cervical cancer cell lines. Expression increases in a time-dependent manner upon exposure to radiation. |
|  |  | Wei et al., 2020 [49] | Upregulated in pancreatic cancer patients’ serum. Serum levels significantly decreased following tumor resection. |
| Hsa-miR-548K | Down-regulated in HPV positive OPC compared to HPV positive controls  Down-regulated in HPV negative OPC compared to HPV negative controls | Lu et al., 2019 [15] | Up-regulation in HNSCC tissue indicate poor prognosis |
|  |  | Zhang et al., 2018 [34] | Down-regulated in HPV positive HNSCC tissue compared to HPV negative HNSCC tissue |
|  |  | Chen et al., 2018 [50] | Upregulated in esophageal squamous cell carcinoma (ESCC) tissue. Promote cell proliferation and migration. Target long non-coding RNA LET. |
|  |  | Zhang et al., 2018 [34] | Upregulated in ESCC tissue. Promote lymphangiogenesis and lymphatic metastasis. |
| Hsa-miR-1246 |  | Bhagirath et al., 2018 [51] | Downregulated in prostate cancer tissue and cell lines. MiR-1246 is selectively exported via exosomes and significantly upregulated in the serum of prostate cancer patients. Inhibited tumor growth, invasion, and migration and promoted apoptosis. |
|  |  | Wang et al., 2016 [52] | Upregulated in CRC tissue and cell lines. Targets CCNG2 and promote proliferation, invasion, and migration and suppress apoptosis. |
|  |  | Zhang et al., 2016 [46] | Upregulated in lung cancer CD166+ tumor-initiating cells, tumor tissue, and patients’ serum. Promote tumorigenesis and metastatic potential. The level is negatively associated with patient survival. Inhibition suppress sphere-formation and tumor growth. |
|  |  | Zhang et al., 2013 [48] | Upregulated in radiotherapy resistant cervical cancer cell lines compared to radiotherapy sensitive cervical cancer cell lines. Expression increases in a time-dependent manner upon exposure to radiation. |

Reference

1. Hou Y, Zhen J, Xu X, Zhen K, Zhu B, Pan R, et al. miR-215 functions as a tumor suppressor and directly targets ZEB2 in human non-small cell lung cancer. Oncology letters. 2015; 10: 1985-92.

2. Ge G, Zhang W, Niu L, Yan Y, Ren Y, Zou Y. miR-215 functions as a tumor suppressor in epithelial ovarian cancer through regulation of the X-chromosome-linked inhibitor of apoptosis. Oncology Reports. 2016; 35: 1816-22.

3. LEBLANC N, HARQUAIL J, CRAPOULET N, OUELLETTE RJ, ROBICHAUD GA. Pax-5 Inhibits Breast Cancer Proliferation Through MiR-215 Up-regulation. Anticancer Research. 2018; 38: 5013-26.

4. Tang X, Shi X, Wang N, Peng W, Cheng Z. MicroRNA-215-3p Suppresses the Growth, Migration, and Invasion of Colorectal Cancer by Targeting FOXM1. Technology in cancer research & treatment. 2019; 18: 1533033819874776-.

5. Deng Y, Huang Z, Xu Y, Jin J, Zhuo W, Zhang C, et al. MiR-215 modulates gastric cancer cell proliferation by targeting RB1. Cancer Letters. 2014; 342: 27-35.

6. Cai S, Weng Y, Miao F. MicroRNA-194 inhibits PRC1 activation of the Wnt/β-catenin signaling pathway to prevent tumorigenesis by elevating self-renewal of non-side population cells and side population cells in esophageal cancer stem cells. Cell and Tissue Research. 2021; 384: 353-66.

7. Wang Y, Yang L, Chen T, Liu X, Guo Y, Zhu Q, et al. A novel lncRNA MCM3AP-AS1 promotes the growth of hepatocellular carcinoma by targeting miR-194-5p/FOXA1 axis. Molecular Cancer. 2019; 18: 28.

8. Wang C, Li X, Zhang L, Chen Y, Dong R, Zhang J, et al. miR-194-5p down-regulates tumor cell PD-L1 expression and promotes anti-tumor immunity in pancreatic cancer. International Immunopharmacology. 2021; 97: 107822.

9. Bai R, Dou K, Wu Y, Ma Y, Sun J. The NF-κB modulated miR-194-5p/IGF1R/PPFIBP axis is crucial for the tumorigenesis of ovarian cancer. J Cancer. 2020; 11: 3433-45.

10. Wang F, Ji X, Wang J, Ma X, Yang Y, Zuo J, et al. LncRNA PVT1 Enhances Proliferation and Cisplatin Resistance via Regulating miR-194-5p/HIF1a Axis in Oral Squamous Cell Carcinoma. OncoTargets and therapy. 2020; 13: 243-52.

11. Veena MS, Raychaudhuri S, Basak SK, Venkatesan N, Kumar P, Biswas R, et al. Dysregulation of hsa-miR-34a and hsa-miR-449a leads to overexpression of PACS-1 and loss of DNA damage response (DDR) in cervical cancer. Journal of Biological Chemistry. 2020; 295: 17169-86.

12. Ye W, Xue J, Zhang Q, Li F, Zhang W, Chen H, et al. MiR-449a functions as a tumor suppressor in endometrial cancer by targeting CDC25A. Oncology Reports. 2014; 32: 1193-9.

13. Bach D-H, Kim D, Bae SY, Kim WK, Hong J-Y, Lee H-J, et al. Targeting Nicotinamide N-Methyltransferase and miR-449a in EGFR-TKI-Resistant Non-Small-Cell Lung Cancer Cells. Molecular Therapy - Nucleic Acids. 2018; 11: 455-67.

14. Noonan EJ, Place RF, Pookot D, Basak S, Whitson JM, Hirata H, et al. miR-449a targets HDAC-1 and induces growth arrest in prostate cancer. Oncogene. 2009; 28: 1714-24.

15. Lu L, Wu Y, Feng M, Xue X, Fan Y. A novel seven‑miRNA prognostic model to predict overall survival in head and neck squamous cell carcinoma patients. Mol Med Rep. 2019; 20: 4340-8.

16. Lajer CB, Garnæs E, Friis-Hansen L, Norrild B, Therkildsen MH, Glud M, et al. The role of miRNAs in human papilloma virus (HPV)-associated cancers: bridging between HPV-related head and neck cancer and cervical cancer. British journal of cancer. 2012; 106: 1526-34.

17. Tsukigi M, Bilim V, Yuuki K, Ugolkov A, Naito S, Nagaoka A, et al. Re-expression of miR-199a suppresses renal cancer cell proliferation and survival by targeting GSK-3β. Cancer Letters. 2012; 315: 189-97.

18. Ye H, Pang L, Wu Q, Zhu Y, Guo C, Deng Y, et al. A critical role of mir-199a in the cell biological behaviors of colorectal cancer. Diagnostic pathology. 2015; 10: 65.

19. Byrnes KA, Phatak P, Mansour D, Xiao L, Zou T, Rao JN, et al. Overexpression of miR-199a-5p decreases esophageal cancer cell proliferation through repression of mitogen-activated protein kinase kinase kinase-11 (MAP3K11). Oncotarget. 2016; 7: 8756-70.

20. Sakaguchi T, Yoshino H, Yonemori M, Miyamoto K, Sugita S, Matsushita R, et al. Regulation of ITGA3 by the dual-stranded microRNA-199 family as a potential prognostic marker in bladder cancer. British Journal of Cancer. 2017; 116: 1077-87.

21. Han L, Sun Y, Lu C, Ma C, Shi J, Sun D. MiR-3614-5p Is a Potential Novel Biomarker for Colorectal Cancer. 2021; 12.

22. Li F, Yang H, Kong T, Chen S, Li P, Chen L, et al. PGAM1, regulated by miR-3614-5p, functions as an oncogene by activating transforming growth factor-β (TGF-β) signaling in the progression of non-small cell lung carcinoma. Cell Death & Disease. 2020; 11: 710.

23. Yin P, Tong C. LncRNA RGMB-AS1 up-regulates ANKRD1 through competitively sponging miR-3614-5p to promote OSA cell proliferation and invasion. Archives of medical research. 2021.

24. Song A, Wu Y, Chu W, Yang X, Zhu Z, Yan E, et al. Involvement of miR-619-5p in resistance to cisplatin by regulating ATXN3 in oral squamous cell carcinoma. International journal of biological sciences. 2021; 17: 430-47.

25. Zheng Y, Song A, Zhou Y, Zhong Y, Zhang W, Wang C, et al. Identification of extracellular vesicles-transported miRNAs in Erlotinib-resistant head and neck squamous cell carcinoma. Journal of Cell Communication and Signaling. 2020; 14: 389-402.

26. Zhang J, Sun W, Ren C, Kong X, Yan W, Chen X. A PolH Transcript with a Short 3′UTR Enhances PolH Expression and Mediates Cisplatin Resistance. 2019; 79: 3714-24.

27. Shkurnikov MY, Makarova YA, Knyazev EN, Fomicheva KA, Galatenko AV, Nyushko KM, et al. Plasma Level of hsa-miR-619-5p microRNA Is Associated with Prostatic Cancer Dissemination beyond the Capsule. Bulletin of Experimental Biology and Medicine. 2017; 163: 475-7.

28. Kim DH, Park S, Kim H, Choi YJ, Kim SY, Sung KJ, et al. Tumor-derived exosomal miR-619-5p promotes tumor angiogenesis and metastasis through the inhibition of RCAN1.4. Cancer Letters. 2020; 475: 2-13.

29. Li Q, Wu X, Guo L, Shi J, Li J. MicroRNA-7-5p induces cell growth inhibition, cell cycle arrest and apoptosis by targeting PAK2 in non-small cell lung cancer. 2019; 9: 1983-93.

30. Yin CY, Kong W, Jiang J, Xu H, Zhao W. miR‑7‑5p inhibits cell migration and invasion in glioblastoma through targeting SATB1. Oncology Letters. 2018.

31. Dong M, Xie Y, Xu Y. miR‑7‑5p regulates the proliferation and migration of colorectal cancer cells by negatively regulating the expression of Krüppel‑like factor 4. Oncol Lett. 2019; 17: 3241-6.

32. Jung HM, Phillips BL, Patel RS, Cohen DM, Jakymiw A, Kong WW, et al. Keratinization-associated miR-7 and miR-21 Regulate Tumor Suppressor Reversion-inducing Cysteine-rich Protein with Kazal Motifs (RECK) in Oral Cancer *<sup></sup>. Journal of Biological Chemistry. 2012; 287: 29261-72.

33. Weng Z, Peng J, Wu W, Zhang C, Zhao J, Gao H. Downregulation of PART1 Inhibits Proliferation and Differentiation of Hep3B Cells by Targeting hsa-miR-3529-3p/FOXC2 Axis. Journal of Oncology. 2021; 2021: 7792223.

34. Zhang W, Hong R, Li L, Wang Y, Du P, Ou Y, et al. The chromosome 11q13.3 amplification associated lymph node metastasis is driven by miR-548k through modulating tumor microenvironment. Molecular cancer; 2018. p. 125.

35. Kinget L, Roussel E, Verbiest A, Albersen M, Rodríguez-Antona C, Graña-Castro O, et al. MicroRNAs Targeting HIF-2α, VEGFR1 and/or VEGFR2 as Potential Predictive Biomarkers for VEGFR Tyrosine Kinase and HIF-2α Inhibitors in Metastatic Clear-Cell Renal Cell Carcinoma. 2021; 13: 3099.

36. Shang Y, Wang L, Zhu Z, Gao W, Li D, Zhou Z, et al. Downregulation of miR-423-5p Contributes to the Radioresistance in Colorectal Cancer Cells. 2021; 10.

37. Okada R, Koshizuka K, Yamada Y, Moriya S, Kikkawa N, Kinoshita T, et al. Regulation of Oncogenic Targets by miR-99a-3p (Passenger Strand of miR-99a-Duplex) in Head and Neck Squamous Cell Carcinoma. Cells. 2019; 8: 1535.

38. Qi C-L, Sheng J-F, Huang M-L, Zou Y, Wang Y-P, Wang F, et al. Integrated analysis of deregulation microRNA expression in head and neck squamous cell carcinoma. Medicine. 2021; 100.

39. Shinden Y, Hirashima T, Nohata N, Toda H, Okada R, Asai S, et al. Molecular pathogenesis of breast cancer: impact of miR-99a-5p and miR-99a-3p regulation on oncogenic genes. Journal of Human Genetics. 2021; 66: 519-34.

40. Luo C, Yin D, Zhan H, Borjigin U, Li C, Zhou Z, et al. microRNA-501-3p suppresses metastasis and progression of hepatocellular carcinoma through targeting LIN7A. Cell Death & Disease. 2018; 9: 535.

41. He L, Chen S, Ying Y, Xie H, Li J, Ma X, et al. MicroRNA-501-3p inhibits the proliferation of kidney cancer cells by targeting WTAP. 2021; 10: 7222-32.

42. Lu J, Zhou L, Wu B, Duan Y, Sun Y, Gu L, et al. MiR-501-3p functions as a tumor suppressor in non-small cell lung cancer by downregulating RAP1A. Experimental Cell Research. 2020; 387: 111752.

43. Wu F, Xing T, Gao X, Liu F. miR‑501‑3p promotes colorectal cancer progression via activation of Wnt/β‑catenin signaling. Int J Oncol. 2019; 55: 671-83.

44. Sanches JGP, Xu Y, Yabasin IB, Li M, Lu Y, Xiu X, et al. miR-501 is upregulated in cervical cancer and promotes cell proliferation, migration and invasion by targeting CYLD. Chemico-biological interactions. 2018; 285: 85-95.

45. Nakashima H, Yoshida R, Hirosue A, Kawahara K, Sakata J, Arita H, et al. Circulating miRNA-1290 as a potential biomarker for response to chemoradiotherapy and prognosis of patients with advanced oral squamous cell carcinoma: A single-center retrospective study. Tumor Biology. 2019; 41: 1010428319826853.

46. Zhang WC, Chin TM, Yang H, Nga ME, Lunny DP, Lim EKH, et al. Tumour-initiating cell-specific miR-1246 and miR-1290 expression converge to promote non-small cell lung cancer progression. Nature Communications. 2016; 7: 11702.

47. Yao T, Rao Q, Liu L, Zheng C, Xie Q, Liang J, et al. Exploration of tumor-suppressive microRNAs silenced by DNA hypermethylation in cervical cancer. Virology Journal. 2013; 10: 175.

48. Zhang B, Chen J, Ren Z, Chen Y, Li J, Miao X, et al. A specific miRNA signature promotes radioresistance of human cervical cancer cells. Cancer Cell International. 2013; 13: 118.

49. Wei J, Yang L, Wu Y-n, Xu J. Serum miR-1290 and miR-1246 as Potential Diagnostic Biomarkers of Human Pancreatic Cancer. J Cancer. 2020; 11: 1325-33.

50. Chen Z, Lin J, Wu S, Xu C, Chen F, Huang Z. Up-regulated miR-548k promotes esophageal squamous cell carcinoma progression via targeting long noncoding RNA-LET. Experimental Cell Research. 2018; 362: 90-101.

51. Bhagirath D, Yang TL, Bucay N, Sekhon K, Majid S, Shahryari V, et al. microRNA-1246 Is an Exosomal Biomarker for Aggressive Prostate Cancer. 2018; 78: 1833-44.

52. Wang S, Zeng Y, Zhou J-M, Nie S-L, Peng Q, Gong J, et al. MicroRNA-1246 promotes growth and metastasis of colorectal cancer cells involving CCNG2 reduction. Molecular Medicine Reports. 2016; 13: 273-80.
